# Understanding PSA and its derivatives in prediction of tumor volume: addressing health disparities in prostate cancer risk stratification

**DOI:** 10.18632/oncotarget.14903

**Published:** 2017-01-30

**Authors:** Felix M Chinea, Kirill Lyapichev, Jonathan I Epstein, Deukwoo Kwon, Paul Taylor Smith, Alan Pollack, Richard J Cote, Oleksandr N Kryvenko

**Affiliations:** ^1^ Department of Radiation Oncology, University of Miami Miller School of Medicine, Miami, FL, USA; ^2^ Pathology and Laboratory Medicine, University of Miami Miller School of Medicine, Miami, FL, USA; ^3^ Urology, University of Miami Miller School of Medicine, Miami, FL, USA; ^4^ Biostatistics, University of Miami Miller School of Medicine, Miami, FL, USA; ^5^ Biochemistry, University of Miami Miller School of Medicine, Miami, FL, USA; ^6^ Sylvester Comprehensive Cancer Center, University of Miami Miller School of Medicine, Miami, FL, USA; ^7^ Departments of Pathology, Urology, and Oncology, The Johns Hopkins Medical Institutions, Baltimore, MD, USA

**Keywords:** prostate cancer, prostate specific antigen, health disparities, Hispanic/Latino, risk stratification

## Abstract

**Objectives:**

To address health disparities in risk stratification of U.S. Hispanic/Latino men by characterizing influences of prostate weight, body mass index, and race/ethnicity on the correlation of PSA derivatives with Gleason score 6 (Grade Group 1) tumor volume in a diverse cohort.

**Results:**

Using published PSA density and PSA mass density cutoff values, men with higher body mass indices and prostate weights were less likely to have a tumor volume <0.5 cm^3^. Variability across race/ethnicity was found in the univariable analysis for all PSA derivatives when predicting for tumor volume. In receiver operator characteristic analysis, area under the curve values for all PSA derivatives varied across race/ethnicity with lower optimal cutoff values for Hispanic/Latino (PSA=2.79, PSA density=0.06, PSA mass=0.37, PSA mass density=0.011) and Non-Hispanic Black (PSA=3.75, PSA density=0.07, PSA mass=0.46, PSA mass density=0.008) compared to Non-Hispanic White men (PSA=4.20, PSA density=0.11 PSA mass=0.53, PSA mass density=0.014).

**Materials and Methods:**

We retrospectively analyzed 589 patients with low-risk prostate cancer at radical prostatectomy. Pre-operative PSA, patient height, body weight, and prostate weight were used to calculate all PSA derivatives. Receiver operating characteristic curves were constructed for each PSA derivative per racial/ethnic group to establish optimal cutoff values predicting for tumor volume ≥0.5 cm^3^.

**Conclusions:**

Increasing prostate weight and body mass index negatively influence PSA derivatives for predicting tumor volume. PSA derivatives’ ability to predict tumor volume varies significantly across race/ethnicity. Hispanic/Latino and Non-Hispanic Black men have lower optimal cutoff values for all PSA derivatives, which may impact risk assessment for prostate cancer.

## INTRODUCTION

From 2005 to 2009, Non-Hispanic Black (NHB) men were reported with a 63% greater incidence and 144% greater mortality from prostate cancer (PCa) than Non-Hispanic White (NHW) men [1]. Not only is this a result of socioeconomic [2] and cultural [3] influences, but biological as well [4–6]. In NHB patients, the tumor burden tends to localize more anteriorly, has an increased volume [5], and is more likely to show progression on re-biopsy during active surveillance (AS) [7]. This has contributed to the reasoning that biopsy and AS criteria should be modified for NHB men [4, 5]. Many screening tools used for determining recommendations for prostate biopsy and appropriateness of AS have yet to be adequately explored in Hispanic/Latino men, the largest minority population in the United States [8]. Despite having a greater relative risk of PCa-specific mortality [9] and recently demonstrating worse radical prostatectomy (RP) outcomes compared to NHW men [10], relatively little is known about the clinicopathologic profile of Hispanic/Latino men in the setting of risk stratification for prostate biopsy and AS.

In their systematic review of clinicopathologic variables and biomarkers for risk stratification of PCa, Loeb *et al*. concluded that PSA-based tests can help in predicting risk of disease progression [11]. While the American Urological Association recommends PSA as the best screening tool in assessing PCa risk level and ultimately selecting patients for biopsy [12], others have discussed factors that modify the sensitivity and specificity of serum PSA, such as body mass index (BMI) and prostate weight (PW) [4, 13–15]. To account for prostate size, PSA density (PSAD) was introduced and is superior to PSA in predicting tumor volume (TV) [4, 16, 17]. A hemodilution effect has also been discussed with overweight and obese men having disproportionally lower serum PSA levels [14]. PSA mass (PSAM) accounts for this effect and also improves correlation with TV, although it does not account for PW [18]. Through incorporation of PSAM and PSAD, PSA mass density (PSAMD) accounts for both PW and BMI, potentially improving correlations with TV compared to PSA or PSAD [15].

PSAD is one of the five factors within the original and modified Epstein AS criteria [4, 19]. With evidence suggesting racial differences in PSA production [6, 20] and in the predictability of AS criteria [4, 5, 10], investigating the use of PSA-based tests in risk stratification of racially and ethnically diverse patient populations becomes increasingly important. To our knowledge, only one study has attempted to explore PSAD in Hispanic/Latino men; that study showed similar PSA with higher PSAD levels amongst Hispanic/Latino compared to NHW men, but did not account for disease severity or BMI [21]. With Hispanic/Latino men being at significantly higher risk of PCa and PCa-specific mortality, characterizing PSA and its derivatives for the prediction of TV in a preoperative setting would provide invaluable guidance on appropriate clinical screening and AS recommendations. We describe the variability of PSA and its derivatives to preoperatively predict TV in a diverse cohort.

## RESULTS

Table 1 shows patient characteristics organized by racial/ethnic groups. In this cohort, 390 (66.2%) men were NHW, 87 (14.8%) were NHB, 78 (13.2%) were Hispanic/Latino, and 34 (5.8%) of other races and/or ethnicities or unknown. Statistically significant differences were found in mean values for PSAD (*p*= 0.04), PSAMD (*p*= 0.04), PW (*p*= 0.002), and originating institution (*p* <0.001) across racial/ethnic groups: NHW, NHB, Hispanic/Latino, and other men. All other clinical and pathological variables were not statistically different. To address any potential regional confounders, Table 2 demonstrates no statistical difference between PSA derivatives, PW, and TV across home institutions. Supplementary Table 1 shows this same cohort divided by BMI categories. There were statistically significant differences found between these groups in PW (p<0.001), PSAM (p= 0.01), and PSAD (p= 0.001). Differences in PSAMD were statistically insignificant (p= 0.60), suggesting its potential for equal assessment of men, regardless of body weight. No significant differences were found between other parameters.

**Table 1:** Patient characteristics sorted by racial/ethnic group

	*All* (n=589)		*Non-Hispanic White* (n=390)		*Non-Hispanic Black* (n=87)		*Hispanic/Latino* (n=78)		*Other* (n=34)		*p-value^a^*
	Mean	Median(Min, Max)	Mean	Median(Min, Max)	Mean	Median(Min, Max)	Mean	Median(Min, Max)	Mean	Median(Min, Max)	
*Age, years*	57.6	58(36, 78)	57.8	58(40, 74)	56.2	57(36, 70)	58.7	59(43, 72)	57.3	57.5(43, 78)	0.16
*PSA, ng/mL*	4.6	4.4(0.3, 13)	4.6	4.4(0.3, 13)	4.8	4.4(0.8, 11.8)	4.4	4.5(0.3, 9.64)	4.3	4.2(1.3, 8.3)	0.69
*PSA density, ng/mL/gm*	0.101	0.098(0.007, 0.284)	0.104	0.101(0.007, 0.282)	0.089	0.081(0.020, 0.257)	0.105	0.095(0.015, 0.284)	0.108	0.098(0.017, 0.232)	0.04
*PSA mass, μg*	0.56	0.53(0.04, 1.76)	0.57	0.54(0.04, 1.76)	0.59	0.55(0.11, 1.30)	0.51	0.50(0.04, 1.18)	0.49	0.49(0.16, 0.93)	0.08
*PSA mass density, μg/gm*	0.012	0.012(0.001, 0.056)	0.013	0.012(0.001, 0.056)	0.011	0.010(0.003, 0.029)	0.012	0.010(0.002, 0.036)	0.012	0.011(0.002, 0.026)	0.04
*Prostate weight, gm*	49.2	44.9(18, 194)	47.9	44.9(19.7, 165.5)	57.9	51.5(24.5, 194)	46.9	42(18, 97)	47.4	41.0(22, 146)	0.002
*Tumor volume, cm^3^*	0.70	0.37(0.004, 7.6)	0.67	0.35(0.004, 6.32)	0.85	0.51(0.01, 7.02)	0.71	0.35(0.03, 7.57)	0.74	0.39(0.03, 2.97)	0.11
	No. (%)		No. (%)		No. (%)		No. (%)		No. (%)		*p-value^b^*
**BMI**^c^											0.22
*Normal*	160 (66.6%)		110 (28.4%)		15 (18.1%)		21 (26.9%)		14 (41.2%)		
*Overweight*	307 (14.2%)		201 (51.8%)		47 (56.6%)		43 (55.1%)		16 (47.1%)		
*Obese*	116 (13.4%)		77 (19.8%)		21 (25.3%)		14 (17.9%)		4 (11.8%)		
**Institution**											<0.001
*JHU*	447 (76.7%)		352 (90.3%)		70 (80.5%)		0 (0.0%)		31 (91.2%)		
*UM*	136 (23.3%)		38 (9.7%)		17 (19.5%)		78 (100.0%)		3 (8.8%)		

Abbreviations: UM = The University of Miami; JHU = The Johns Hopkins University.

^a^ Kruskal-Wallis test was used in calculation of p-values for continuous variables.

^b^ Chi-square test was used in calculation of p-values for categorical variables.

^c^ Six patients had missing BMI values.

**Table 2:** Patient characteristics sorted by institution

	*UM* (n=136)		*JHU* (n=453)		*p-value^a^*
	Mean	Median(Min, Max)	Mean	Median(Min, Max)	
*Age (years)*	59.6	60(40, 7)	57.0	57(36, 73)	<0.001
*PSA, ng/mL*	4.76	4.55(0.3, 13)	4.56	4.4(0.4, 10.1)	0.63
*PSA density, ng/mL/gm*	0.10	0.096(0.012, 0.284)	0.10	0.099(0.007, 0.257)	0.60
*PSA mass, μg*	0.56	0.53(0.03, 1.61)	0.56	0.53(0.05, 1.78)	0.85
*PSA mass density, μg/gm*	0.01	0.011(0.001, 0.036))	0.01	0.012(0.0009, 0.056)	0.27
*Prostate weight, gm*	50.6	45(18, 146)	48.8	44.8(19.7, 194)	0.43
*Tumor volume, cm^3^*	0.75	0.35(0.004, 7.57)	0.69	0.38(0.003, 6.32)	0.92
	No. (%)		No. (%)		*p-value^b^*
**Race/ethnicity**					<0.001
*Non-Hispanic White*	38 (27.9%)		352 (77.7%)		
*Non-Hispanic Black*	17 (12.5%)		70 (15.5%)		
*Hispanic/Latino*	78 (57.4%)		-		
*Other*	3 (2.2%)		31 (6.8%)		
**BMI**^c^					0.47
*Normal*	35 (25.7%)		125 (28.0%)		
*Overweight*	69 (50.7%)		238 (53.2%)		
*Obese*	32 (23.6%)		84 (18.8%)		

Abbreviations: UM = The University of Miami; JHU = The Johns Hopkins University.

^a^ Kruskal-Wallis test was used in calculation of p-values for continuous variables.

^b^ Chi-square test was used in calculation of p-values for categorical variables.

^c^ Six patients had missing BMI values.

**Figure 1:**
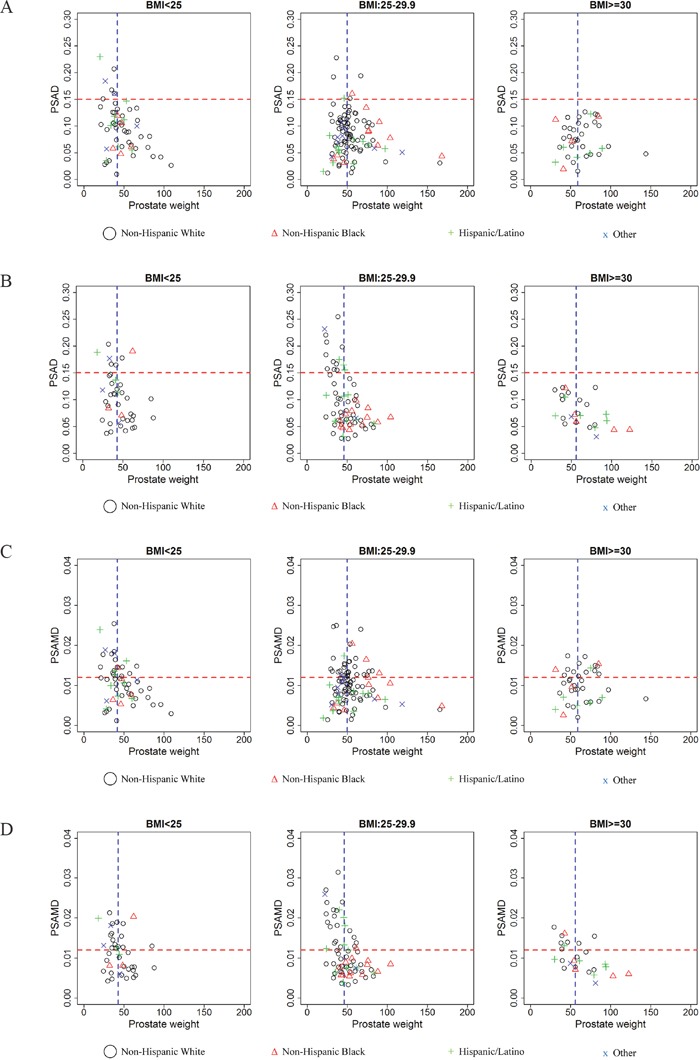
Potential PSA density (PSAD) screening of men with tumor volume **A**. <0.5 cm^3^ and **B**. ≥0.5 cm^3^ as well as potential PSA mass density (PSAMD) screening of men with tumor volume: **C**. <0.5 cm^3^ and **D**. ≥0.5 cm^3^. Both groups are sorted by body mass index (BMI) categories: normal weight (BMI <25), overweight (BMI= 25-29.9, and obese (BMI >30). Cutoff values (horizontal red line) are displayed to indicate those identified by screening test (PSAD >0.15 and PSAMD >0.012) and median prostate weight (vertical blue line) for each group is displayed to compare relatively smaller and larger prostate glands. Race and/or ethnicity is indicated for Non-Hispanic White (black circle), Non-Hispanic Black (red triangle), Hispanic/Latino (green cross), and other men (blue x). Those above the cutoff values with <0.5 cm^3^ (A) and (C) are considered false positive results, while those above the cutoffs with ≥0.5 cm^3^ (B) and (D) are considered true positives.

To help visualize the influence of both PW and BMI, Figure 1 displays patients subdivided by TV <0.5 cm^3^and ≥0.5 cm^3^, and divided by BMI category in scatter plots of PW vs. PSAD and PW vs. PSAMD. Cutoff values are based on the Epstein AS criteria [4] (PSAD <0.15) and a previous publication (PSAMD <0.012) [15]. The median PW was used to objectively divide those with relatively smaller from those with relatively larger prostate sizes. These plots demonstrate that with higher BMI values, fewer men are identified through screening with either PSA derivative. While the PSAD cutoff did not identify any obese men with significant disease, PSAMD seemed to perform more consistently across normal weight, overweight, and obese men. Regardless of BMI, these plots also suggest that men with larger PW (greater than the median) are less likely to be identified on screening with PSAD and PSAMD than those with smaller PW (less than the median). Incidentally, regardless of BMI or PW, both NHB and Hispanic/Latino men are less likely to be identified on screening with these measures. We provide more details of this analysis in Supplementary Tables 2 and 3.

**Table 3:** Calculated PSA derivative optimal cutoff values and resulting sensitivity and specificity divided by race/ethnicity

		Cutoff value^a^	Sensitivity (%)	Specificity (%)	PPV(%)	NPV(%)	AUC (%)
PSA, ng/mL							
	*NHW*	4.20	51.3	66.9	52.8	29.7	58.5
	*NHB*	3.75	48.8	75	40	34.4	56.4
	*Hispanic/Latino*	2.79	35.4	90	53.4	15	64.4
PSA density, ng/mL/gm							
	*NHW*	0.11	68.5	62.3	43.5	26.5	70.5
	*NHB*	0.07	60.5	84.1	31.5	21.2	72.6
	*Hispanic/Latino*	0.06	47.9	93.3	47.2	8	75.2
PSA mass, μg							
	*NHW*	0.53	54.7	64.7	51.9	29.5	59.7
	*NHB*	0.46	43.9	78.6	41.1	33.3	55.6
	*Hispanic/Latino*	0.37	41.7	86.7	51.9	16.7	64.9
PSA mass density, μg/gm							
	*NHW*	0.014	75.7	58.2	39	26.4	72.1
	*NHB*	0.008	53.7	88.1	33.9	18.5	73.1
	*Hispanic/Latino*	0.011	68.8	73.3	40.5	19.5	74.6

Abbreviations: NHW = Non-Hispanic White; NHB = Non-Hispanic Black; PPV = positive predictive value; NPV = negative predictive value; AUC = area under the receiver operating characteristic (ROC) curve.

^a^Only NHW, NHB, and Hispanic/Latino men were included in ROC analysis to determine racial/ethnic specific cutoff values.

**Figure 2:**
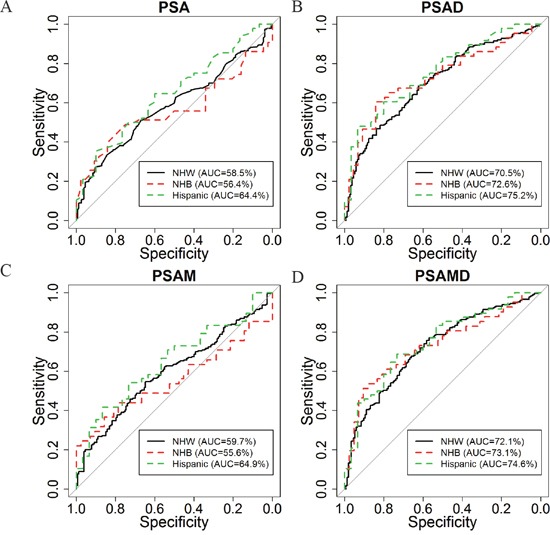
Receiver operating characteristic (ROC) curves of A. PSA, B. PSAD, C. PSA mass (PSAM), and D. PSAMD in discrimination between tumor volume < vs. ≥0.5 cm^3^ for: Non-Hispanic White (black), Non-Hispanic Black (red), and Hispanic/Latino (blue)

**Figure 3:**
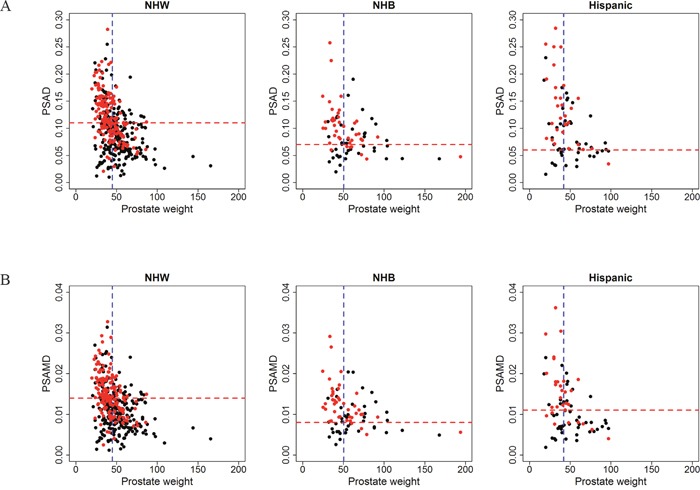
Potential screening tests using calculated optimal cutoff values of A. PSAD and B. PSAMD for Non-Hispanic White (NHW), Non-Hispanic Black (NHB), and Hispanic/Latino men In these plots, men with tumor volume <0.5 cm^3^ (black dot) and men with tumor volume ≥0.5 cm^3^ (red dot) are displayed to visualize performance of racial/ethnic specific cutoff values (horizontal red line) while indicating median prostate weight (vertical blue line) of each group. Black dots or men with <0.5 cm^3^ above the cutoff value are considered false positive results and red dots or men with ≥0.5 cm^3^ are considered true positives.

In an effort to further explore racial/ethnic differences suggested in preliminary correlations and the univariable analysis (Supplementary Table 4), we constructed ROC curves for the ability of PSA, PSAD, PSAM, and PSAMD to predict dichotomized TV values (<0.5 cm^3^ vs. ≥0.5 cm^3^) in each racial/ethnic group (Figure 2). Area under the curve values (AUC) for these PSA derivatives were greatest in PSAD and PSAMD with better performance in Hispanic/Latino and NHB men. Both PSA and PSAM performed similarly, with better performance in Hispanic/Latino men and worst performance in NHB men. Overall, PSAMD results in a higher sensitivity with a fixed specificity of 80%, of statistical significance (*p*= 0.035). In Table 3, we show optimal cutoff values for all PSA-based tests determined by the ROC curves (the most upper left point on the curve) as well as the resulting sensitivity, specificity, positive predictive values, and negative predictive values in each racial/ethnic group. As shown in this table, optimal cutoff values in prediction of dichotomized TV were lowest for Hispanic/Latino (PSAD=0.06 and PSAMD=0.011) and NHB (PSAD=0.07 and PSAMD=0.008) men, while differences in pathologically confirmed TV were not significant across race/ethnicity. To visualize the potential screening performance of these optimal cutoff values, Figure 3 displays each race/ethnicity in scatter plots of PSAD vs. PW and PSAMD vs. PW. These plots demonstrate the decrease in false negative results for NHB and Hispanic/Latino men through the use of race/ethnicity specific cutoff values.

## DISCUSSION

The Hispanic/Latino community constitutes about 17.4% of the U.S. population and has a projected growth to reach 29% by 2050 [8]. Although PCa is the most commonly diagnosed non-cutaneous malignancy in Hispanic/Latino men [22], relatively little is known about low-risk disease in this population. Studies describing biological differences in tumor volume, location [5], PSA production [20], and performance of AS criteria [4] in NHB men provide useful information to enact meaningful changes in clinical screening and management of PCa. Despite recently reported inconsistencies in AS performance across NHW, NHB, and Hispanic/Latino men who underwent RP [4, 5, 10], AS criteria do not yet include race or ethnicity as variables.

In our cohort, we explored the ability of PSA and its derivatives to predict TV. In validation of previous findings regarding the influence of PW on PSA levels, PSAD and PSAMD performed best in both the univariable and the age-adjusted multivariable analysis. As demonstrated in Figure 1, PSAMD can potentially improve the identification of significant disease for overweight and obese men [15]. With Hispanics/Latinos being 1.2 times and NHBs being 1.5 times as likely to be obese compared to NHWs [23], adjusting this biochemical measure for BMI becomes increasingly relevant. Our study reiterates previous findings of PW and BMI as influential factors of PSA in a cohort more reflective of the modern U.S. population.

Variability in PSA production amongst racial/ethnic groups can be a result of biological or environmental factors. In recent findings, while accounting for TV, PW, and BMI, NHB men were shown to produce equal amounts of PSA from benign prostate tissue but 25% less from GS 3+3=6 (Grade Group 1) PCa compared to NHW men [20]. By further characterizing PSA-based tests in minority populations with homogenous, GS 3+3=6 (Grade Group 1) PCa, we can attempt to address risk stratification disparities by isolating and identifying contributing factors. As shown in the univariable analysis, each racial/ethnic group exhibited significant correlations with PSAD and PSAMD predicting for TV. After construction of ROC curves in prediction of the dichotomized TV values (<0.5 cm^3^ vs. ≥0.5 cm^3^), we found variability in both the AUC values and subsequent optimal cutoff values across different racial/ethnic groups. With determined optimal cutoff values for PSAD and PSAMD being lower in Hispanic/Latino and NHB men, plus Figure 3 showing a decrease in false negatives with these values, it suggests that race and ethnicity should be taken into account for screening and AS guidelines. These racial/ethnic specific nuances of PSA production by PCa will be used in combination with clinical stage, cancer extent at biopsy, and potential multiparametric MRI findings to further improve risk stratification of PCa.

Although use of a select group of men with GS 3+3=6 (Grade Group 1) PCa may be a source of limitation, as previously explained, this was to represent a population targeted for AS and to avoid the variability in PSA production exhibited by other Gleason patterns [24, 25]. By accounting for such variability, we were able to define a consistent relationship between PSA production and TV. Although the inclusion of intermediate and high-risk patients would be of great clinical interest, the consistency in PSA production from our cohort allows for the proposal of more reliable PSA-derived cutoff values predicting for significant low-risk PCa TV. These PSA-derived values should be evaluated in future prospective trials, where the same derivatives should be tested in prediction of TV in Gleason score 3+4=7 (Grade Group 2) and higher grade PCa. Although we have combined the data from the two large institutions, a detailed pathological re-review was performed, cases were included consecutively to limit selection bias, an adjustment factor for PW developed on the larger number of cases from the same institutions was utilized, and we controlled for BMI through the use of PSAMD which may account for some geographic variability. Moreover, we have demonstrated that the studied variables did not significantly differ between these cohorts based on geographical location (Table 2). Although our cohort of NHW men was noticeably larger than NHB and Hispanic/Latino men, probably due to an overall lesser amount of these minorities treated with RP and lower incidence of low-risk disease, we were able to reach statistically significant results controlling for potential confounders. Another limitation is the lack of detailed ethnicity for our cohort. Information regarding region of origin or ancestry would be useful in determining whether significant Hispanic/Latino ethnic heterogeneity would have an influence on PSA production and calculated derivatives. For this study, we calculated PSAD and PSAMD using the PW without the seminal vesicles (SVs). Although this measure is not available preoperatively, nearly perfect correlations between the estimated PSAD (calculated by transrectal ultrasound measurements) and actual PSAD (calculated by pathological measurements without SVs) have been demonstrated by Epstein *et al*. [19] (r=0.95) and are would likely be improved with contemporary radiologic techniques. Additionally, men of NCCN very low-risk while using race/ethnicity as an adjustment for the threshold of PSA and its derivatives should be further investigated to explore if such approach could better predict those qualifying for AS across different racial/ethnic groups.

As others have noted racial and ethnic differences in PSA and PSAD production [20, 21], our findings only attempt to further understand the applicability of PSA-related screening models for all U.S. at-risk communities. In future studies, more specific Hispanic/Latino subgroup (e.g. Mexican American, Puerto Rican, Cuban American, etc.) information should be collected, if possible, to explore potential variability within Hispanic/Latino men and more accurately determine biological influences on PSA production. In South Florida, 63% of Hispanic/Latino men diagnosed with PCa are of Cuban origin or ancestry [26]. With our entire Hispanic/Latino cohort coming from the University of Miami, questions regarding the biological influence of African ancestry in predominantly Caribbean-Hispanic/Latino communities and its potential role in tumor location, volume, and aggressiveness should be explored in separate cohorts. Differences in correlations across race/ethnicity should be further explored in larger cohorts with a broader range of GSs to help refine optimal cutoff values for screening and AS decisions in these populations.

In conclusion, PW and BMI are influential factors that interfere with the correlations of serum PSA and its derivatives with TV. PSA and its derivatives show significant differences in prediction of TV across racial/ethnic groups. Our study suggests the need for lower cutoff values in PSA-based tests for Hispanic/Latino and NHB men to potentially improve risk stratification of PCa in these rapidly growing minority communities.

## MATERIALS AND METHODS

### Patient cohort

We retrospectively collected and analyzed 589 consecutive patients with Gleason score (GS) 3+3=6 (Grade Group 1) PCa at RP from The Johns Hopkins Hospital (n=453; 2009-2013) and The University of Miami (n=136; 2010-2015). The collection of these data was done with approval by the institutional review boards of both participating hospitals. All prostate glands were entirely submitted for histological evaluation. We restricted our study to cases with GS 3+3=6 (Grade Group 1) PCa due to greater homogeneity of PSA production compared to higher grade patterns [27]. With GS 3+3=6 (Grade Group 1) PCa having relatively no metastatic potential, PSA production was likely derived from localized PCa and benign prostate tissue. Also, men with GS 3+3=6 (Grade Group 1) PCa at biopsy would most likely be considered for AS, in which accurately predicting TV is clinically important [4, 28].

### Prostate cancer specifications

All specimens were re-reviewed by one urologic pathologist (ONK) and graded according to the most contemporary PCa grading criteria [29, 30]. The TV was determined by mapping PCa on histologic slides that were photocopied in a 1-square-millimeter grid background and each mm^2^ was manually counted. For conversion into mm^3^, the total number of mm^2^ was multiplied by 3 (thickness of the prostate tissue sections) and 1.12 (fixation shrinkage factor), as has been previously described and validated [15, 16, 20]. A TV threshold of 0.5 cm^3^ was considered as significant PCa for our dichotomized analysis based on a recent finding that men with contemporarily graded GS 3+3=6 (Grade Group 1) PCa and TV ≥0.5 cm^3^ were significantly more likely to have extraprostatic extension and positive surgical margin at RP [28]. This TV threshold was also included in the definition of both the original [19] and modified [4] Epstein AS criteria.

### PSA derivative calculations

PSAD was determined by dividing preoperative serum PSA level by PW without SVs [4, 5, 19, 31]. For PSAM, plasma volume was multiplied by the preoperative serum PSA level [18]. The plasma volume was determined by multiplying the estimated body surface area (m^2^) by a 1.67 adjustment factor. Body surface area was determined through the following formula: body weight (kg)^0.425^ x height (m)^0.72^ x 0.007184 [16]. Body weight and height were obtained from the electronic medical record, based on preoperative clinical measurements. PSAMD was calculated by dividing PSAM by PW without SVs [15, 31]. In accordance with the World Health Organization classification system, BMI was categorized: underweight (BMI < 18.5), normal weight (BMI 18.5 – 24.99), overweight (BMI 25-29.99), and obese (BMI ≥ 30).

### National Hispanic Identification Algorithm (NHIA)

To verify the accuracy of documented ethnicity in the medical record, this variable was confirmed through a standardized algorithm established by the North American Association of Central Cancer Registries, the National Hispanic Identification Algorithm (NHIA) [10, 32]. NHIA systematically classifies surnames as heavily, generally, moderately, occasionally, or rarely Hispanic based on the 1990 U.S. Census Spanish Surname List. All surnames categorized as “heavily” Hispanic were coded as Hispanic and all others were coded as Non-Hispanic. NHIA uses race information to eliminate candidates with Spanish surnames who identify as Asian, American Indian, Aleutian, Eskimo, Filipino, Pacific Islander, or Hawaiian. The NHIA algorithm was used in SAS 9.4 for Windows.

### Statistical analysis

Student t-test and chi-square test were used for comparing groups in continuous and categorical variables, respectively. In Supplementary Table 5, analysis of correlations between TV and PSA derivatives were conducted using Pearson's correlation coefficient. Partial correlation coefficient was used when the effect of age was removed. Univariable (Supplementary Tables 4 and 5) and multivariable (Supplementary Table 6) linear regression models were performed to examine the relationship between TV and four PSA-based tests individually. Receiver operating characteristic (ROC) analysis was performed to predict for dichotomized TV values below or above the threshold of significant volume GS 3+3=6 (Grade Group 1) PCa (0.5 cm^3^) [28]. P-values ≤0.05 were considered significant. All analyses were performed using R software (version 3.1.1; R Foundation for Statistical Computing, Vienna, Austria).

## SUPPLEMENTARY MATERIALS FIGURES AND TABLES



## Abbreviations

NHB: Non-Hispanic Black
NHW: Non-Hispanic White
PSA: prostate specific antigen
PCa: prostate cancer
AS: active surveillance
RP: radical prostatectomy
BMI: body mass index
PW: prostate weight
PSAD: PSA density
TV: tumor volume
PSAM: PSA mass
PSAMD: PSA mass density
SVs: seminal vesicles
ROC: receiver operating characteristic.
