# Supplementary material for: Understanding PSA and its derivatives in prediction of tumor volume: addressing health disparities in prostate cancer risk stratification
Source: Oncotarget. 2017 Jan 30;8(13):20802–12. doi: 10.18632/oncotarget.14903 (PMC5400546; doi:10.18632/oncotarget.14903)
Supplement: Supplementary file 1 [file oncotarget-08-20802-s001.pdf]

Understanding PSA and its derivatives in prediction of tumor volume: addressing health disparities in prostate cancer risk stratification

SUPPLEMENTARY TABLES

Supplementary Table 1: General characteristics of men at different BMI categories

Variable	**Normal weight (BMI < 25)**	**Overweight (BMI 25-<30)**	**Obese (BMI ≥30)**	*p-value*[a]
	**n=160**	**n=307**	**n=116**	
	**Mean ± SD**	**Mean ± SD**	**Mean ± SD**	
BMI[b]	23.1 ± 1.56	27.5 ± 1.44	33.0 ± 2.64	n/a
Patient height, m	1.78 ± 0.07	1.78 ± 0.07	1.78 ± 0.07	0.87
Prostate weight, gm	43.5 ± 15.4	49.7 ± 21.9	55.2 ± 20.8	<0.001
PSA, ng/mL	4.5 ± 1.9	4.6 ± 2.0	4.7 ± 2.0	1.0
PSA mass, µg	0.51 ± 0.21	0.56 ± 0.25	0.62 ± 0.27	0.01
PSA density, ng/mL/gm	0.11 ± 0.05	0.10 ± 0.05	0.09 ± 0.04	0.001
PSA mass density, µg/gm	0.012 ± 0.01	0.012 ± 0.01	0.012 ± 0.01	0.60
Tumor volume, cm^3	0.65 ± 0.89	0.67 ± 0.88	0.84 ± 1.16	0.82

[a] Kruskal-Wallis test was used in calculation of p-values.
[b] Six patients had missing BMI values and were excluded from this table.

Supplementary Table 2: Comparison of clinical and pathological parameters in patients with insignificant tumor volume in different BMI categories dichotomized according to gland weight

Tumor volume <0.5 cm³													
	Normal weight (BMI < 25), n=98				Overweight (BMI 25-29), n=178				Obese (≥30), n=67				
	[a]Median PW = 42.2				[a]Median PW = 49.1				[a]Median PW = 58				*p-value*[b]
	All (n=98)	< MPW (n=49)	≥ MPW (n=49)		All (n=178)	< MPW (n=89)	≥ MPW (n=89)		All (n=67)	< MPW (n=32)	≥ MPW (n=35)		
	Mean ± SD	Mean ± SD		*p-value*[c]	Mean ± SD	Mean ± SD		*p-value*[c]	Mean ± SD	Mean ± SD		*p-value*[c]	
BMI	23.1 ± 1.48	23.0 ± 1.7	23.3 ± 1.3	0.56	27.5 ± 1.4	27.5 ± 1.5	27.5 ± 1.3	0.77	33.0 ± 2.7	33.1 ± 2.3	32.8 ± 3.0	0.28	<0.001
Patient height, m	1.78 ± 0.06	1.77 ± 0.06	1.78 ± 0.1	0.39	1.78 ± 0.07	1.78 ± 0.06	1.78 ± 0.07	0.63	1.78 ± 0.07	1.78 ± 0.07	1.76 ± 0.07	0.40	0.78
Prostate weight, gm	46.3 ± 1.75	33.3 ± 6.37	59.3 ± 14.9	<0.001	52.8 ± 21.3	38.4 ± 7.3	67.3 ± 21.0	<0.001	62.2 ± 22.2	44.6 ± 8.0	78.3 ± 18.5	<0.001	<0.001
Tumor volume, cm³	0.18 ± 0.13	0.19 ± 0.1	0.18 ± 0.1	0.6	0.18 ± 0.2	0.19 ± 0.2	0.16 ± 0.15	0.37	0.18 ± 0.1	0.21 ± 0.15	0.15 ± 0.1	0.19	0.52
PSA, ng/mL	4.3 ± 1.98	3.8 ± 1.8	4.8 ± 2.0	0.03	4.4 ± 2.1	3.6 ± 1.9	5.1 ± 2.1	<0.001	4.7 ± 2.3	3.5 ± 1.2	5.8 ± 2.4	<0.001	0.72
PSA mass, µg	0.48 ± 0.22	0.42 ± 0.2	0.54 ± 0.2	0.02	0.53 ± 0.3	0.44 ± 0.2	0.62 ± 0.3	<0.001	0.61 ± 0.3	0.46 ± 0.2	0.74 ± 0.3	<0.001	0.03
PSA density, ng/mL/gm	0. 10 ± 0.05	0.11 ± 0.1	0.08 ± 0.04	0.003	0.09 ± 0.05	0.1 ± 0.1	0.08 ± 0.03	0.08	0.08 ± 0.03	0.08 ± 0.03	0.08 ± 0.03	0.51	0.02
PSA mass density, µg/gm	0.011 ± 0.01	0.013 ± 0.01	0.009 ± 0.004	0.002	0.011 ± 0.01	0.012 ± 0.01	0.01 ± 0.004	0.10	0.009 ± 0.003	0.01 ± 0.003	0.009 ± 0.004	0.32	0.52
PSA density > 0.15	14/98 (14%)	12/49	2/49	0.01	20/178 (11%)	17/87	3/91	0.001	0/67 (0%)	0/32	0/35	n/a	0.01
PSA mass density > 0.012	40/98 (41%)	29/49	11/49	<0.001	60/178 (34%)	35/87	25/91	0.10	25/67 (36%)	12/32	13/35	0.99	0.50

Abbreviations: BMI = body mass index; PW = prostate weight; MPW = median prostate weight.
[a] prostates are dichotomized at a threshold of median prostate weight at each BMI category.
[b] p-value applicable to general column (Kruskal-Wallis test for continuous and Fisher test for categorical variables).
[c] p-value comparing distribution of findings in each BMI category.
Wilcoxon-Mann-Whitney rank sum test (U test) was used for comparison of 2 sets of data.

Supplementary Table 3: Comparison of clinical and pathological parameters in patients with significant tumor volume in different BMI categories dichotomized according to gland weight

	Tumor volume ≥0.5 cm³												
	Normal weight (BMI < 25), n=62				Overweight (BMI 25-29), n=127				Obese (≥30), n=49				
	[a]Median PW = 38.6				[a]Median PW = 40.2				[a]Median PW = 42.0				*p-value*[b]
	All (n=62)	< MPW (n=31)	≥ MPW (n=31)		All (n=127)	< MPW (n=64)	≥ MPW (n=63)		All (n=49)	< MPW (n=24)	≥ MPW (n=25)		
	Mean ± SD	Mean ± SD		*p-value*[c]	Mean ± SD	Mean ± SD		*p-value*[c]	Mean ± SD	Mean ± SD		*p-value*[c]	
BMI	22.9 ± 1.7	22.8 ± 1.6	23.1 ± 1.8	0.31	27.5 ± 1.5	27.2 ± 1.4	27.8 ± 1.5	0. 06	33.0 ± 2.6	32.9 ± 3.2	33.0 ± 2.1	0.29	<0.001
Patient height, m	1.78 ± 0.08	1.76 ± 0.07	1.80 ± 0.08	0.07	1.78 ± 0.1	1.77 ± 0.07	1.80 ± 0.07	0.10	1.80 ± 0.1	1.79 ± 0.1	1.80 ± 0.05	0.63	0.41
Prostate weight, gm	39.2 ± 10.5	30.6 ± 4.5	47.7 ± 7.4	<0.001	45.3 ± 22.1	31.9 ± 5.1	58.9 ± 24.3	<0.001	45.7 ± 14.2	35.2 ± 5.0	56.2 ± 12.5	<0.001	0.06
Tumor volume, cm³	1.38 ± 1.1	1.36 ± 0.8	1.41 ± 1.3	0.67	1.37 ± 1.0	1. 41 ± 1.1	1.3 ± 0.9	0.70	1.73 ± 1.3	1.54 ± 1.0	1.92 ± 1.6	0.60	0.45
PSA, ng/mL	4.9 ± 1.6	4.3 ± 1.4	5.5 ± 1.6	0.01	5.0 ± 1.8	4.6 ± 1.8	5.3 ± 1.7	0.02	4.7 ± 1.5	4.3 ± 1.5	5.0 ± 1.4	0.03	0.60
PSA mass, µg	0.55 ± 0.18	0.47 ± 0.2	0.63 ± 0.2	0.001	0.6 ± 0.2	0.56 ± 0.2	0.65 ± 0.2	0.01	0.64 ± 0.3	0.55 ± 0.2	0.67 ± 0.2	0.03	0.29
PSA density, ng/mL/gm	0.13 ± 0.04	0.14 ± 0.05	0.12 ± 0.03	0.02	0.12 ± 0.05	0.15 ± 0.05	0.1 ± 0.04	<0.001	0.11 ± 0.04	0.12 ± 0.04	0.09 ± 0.03	0.02	0.02
PSA mass density, µg/gm	0.014 ± 0.004	0.016 ± 0.005	0.013 ± 0.003	0.04	0.015 ± 0.006	0.018 ± 0.006	0.012 ± 0.005	<0.001	0.015 ± 0.008	0.016 ± 0.005	0.012 ± 0.004	0.02	0.86
PSA density > 0.15	19/62 (31%)	14/31	5/31	0.03	32/127 (25%)	25/63	7/64	<0.001	6/49 (0.1%)	5/24	1/25	0.17	0.07
PSA mass density > 0.012	44/62 (71%)	23/31	21/31	0.78	80/127 (63%)	52/63	28/64	0.10	33/49 (67%)	19/24	14/25	0.15	0.54

Abbreviations: BMI = body mass index; PW = prostate weight; MPW = median prostate weight.
[a] prostates are dichotomized at a threshold of median prostate weight at each BMI category.
[b] p-value applicable to general column (Kruskal-Wallis test for continuous and Fisher test for categorical variables).
[c] p-value comparing distribution of findings in each BMI category.
Wilcoxon-Mann-Whitney rank sum test (U test) was used for comparison of 2 sets of data.

Supplementary Table 4: Univariable linear regression models to examine PSA derivatives in predicting for total tumor volume within racial/ethnic groups

Variable	*All (n=589)*		*NHW (n=390)*		*NHB (n=87)*		*Hispanic/Latino (n=78)*		*Other (n=34)*	
	R-square	*p-value*	R-square	*p-value*	R-square	*p-value*	R-square	*p-value*	R-square	*p-value*
PSA, ng/mL	0.030	<0.001	0.018	0.01	0.027	0.08	0.078	0.01	0.022	0.40
PSA density, ng/mL/gm	0.098	<0.001	0.090	<0.001	0.152	<0.001	0.176	<0.001	0.148	0.03
PSA mass, μg	0.032	<0.001	0.021	0.003	0.030	0.06	0.080	0.01	0.016	0.48
PSA mass density, μg/gm	0.107	<0.001	0.099	<0.001	0.164	<0.001	0.187	<0.001	0.148	0.03

Abbreviations: NHW = Non-Hispanic White; NHB = Non-Hispanic Black.

R-square denotes a goodness-of-fit measure of linear regression model. P-value is reported for regression coefficient of PSA and its derivatives. These results are based on linear regression models using log-transformed total tumor volume and PSA and its derivatives.

Supplementary Table 5: Analysis of correlation between tumor volume of prostate cancer and PSA and its derivatives

**Variable**	***All***[a]		***All***[b]	***Non-Hispanic White***[b]	***Non-Hispanic Black***[b]	***Hispanic/Latino***[b]	***Other***[b]
	**r**	***p-value***	**r**	**r**	**r**	**r**	**r**
PSA, ng/mL	0.172	<0.001	0.177	0.142	0.194	0.300	0.194
PSA density	0.319	<0.001	0.316	0.303	0.402	0.432	0.184
PSA mass	0.178	<0.001	0.182	0.152	0.204	0.303	0.192
PSA mass density	0.334	<0.001	0.330	0.318	0.417	0.445	0.188

Abbreviations: r = Pearson's correlation coefficient.

[a] Partial correlation coefficient with adjustment of age.

[b] Unadjusted correlation coefficient using log-transformed value.

Supplementary Table 6: Multivariable analysis of PSA derivatives prediction of tumor volume at radical prostatectomy

Variable	R-square	*p-value*
PSA, ng/mL	0.035	<0.001
PSA density	0.115	<0.001
PSA mass	0.037	<0.001
PSA mass density	0.125	<0.001

*Age was included in the model for the adjustment.
R-square denotes a goodness-of-fit measure of linear regression model. p-value is reported for regression coefficient of PSA and its derivatives. These results are based on linear regression models using log-transformed total tumor volume and PSA and its derivatives.
